# Persuasive Technologies and Social Interactions in Professional Environments: Embedded Qualitative Case Study

**DOI:** 10.2196/32613

**Published:** 2022-02-23

**Authors:** Barb Marcolin, Chad Saunders, Benoit Aubert

**Affiliations:** 1 Faculty of Management The University of British Columbia, Okanagan Campus Kelowna, BC Canada; 2 Haskayne School of Business University of Calgary Calgary, AB Canada; 3 Department of Information Technologies HEC Montreal Montreal, QC Canada

**Keywords:** persuasive technology, patient experience platforms, group effects, professional work management, services co-design, self-management, health and wellness outcomes, social environments, work influence

## Abstract

**Background:**

Although previous studies have highlighted the impact of interactions on the web in the context of patient–health care professional (HCP) dyads, this paper extends that context to a triad that includes the role of employers and associated settings with social groups.

**Objective:**

This study aims to evaluate how the interactions between individuals and the social use of the platform affect individuals’ use of persuasive technology and, in turn, their work environment actions and responses, by implementing a persuasive technology health and wellness platform in a work environment.

**Methods:**

For 8 months, we deployed a persuasive technology platform with different combinations of health-related features and content in 1 embedded case design with 8 fire stations for a small Canadian city (total number of participant firefighters, n=141) assigned to 1 of 2 treatments—interactive or static webpages. We used text-based content analysis techniques for outcome measures, drawn from a total of 29 participant exit interviews. In addition, medical assessments were conducted at baseline, midpoint, and end point by 7 HCPs and 1 researcher (BM), who also served as the data steward and managed the study.

**Results:**

Our results reveal that group, social, and work influences introduce new elements to the use of persuasive technology, which interact to foster higher levels of individual success. The platform in our study served as part of a larger social system, providing information that facilitated new behaviors at work and home. The 8-month group programs centered on exercise, nutrition, and smoking cessation. Groups of participants coached by certified professionals showed significant increases in sodium awareness, levels of actual exercise, and consistency of activities. As a result of the study, of 141 people, 15 (10.6%) were notified of serious medical health issues and 29 (20.6%) underwent blood work assessments and a privacy shield (protected by federal law) was enacted to protect employees from losing their employment based on any health concerns disclosed.

**Conclusions:**

The persuasive technology platform, in combination with self-management and professional management and social interactions, significantly altered work management behaviors. Interactions among individual outcomes, group influences, and social situations strongly influenced individuals’ behaviors in their work and home environments. Three things further improved the positive results that we observed: privacy shields (which allowed employees to reveal health concerns without fear of professional consequences), individual private activities aligned with group activities, and integration between HCP work with localized, organizational work roles.

## Introduction

### Background

A wide array of health initiatives rely on the use of information technology to improve health outcomes [1-4]. One area where these technologies show continued promise is facilitating self-monitoring and feedback at work and home with the use of social systems.

Although individual use of persuasive technology platforms has been extensively explored in a health care setting and well summarized in a recent exemplar study [1], far less is known about the use of persuasive technology (1) within a broader work context and (2) in integration with social and professional medical care teams [1,5-8].

Persuasive systems and technologies are generally designed to aid and motivate people to adopt good behaviors [5], while avoiding behaviors that are detrimental to themselves or their community [6]. Other related work can be seen in a recent literature review [9] where many define persuasive systems and technologies as the set of “information technology systems and services developed to change [these] users’ attitudes and behaviours or both” [10] for people to use better in organizations. In order for persuasive technology [9] to be most effective and foster healthy lifestyles, users must be in the right mood, time, and place to receive feedback [11].

In outlining underlying principles of their framework for designing persuasive systems, Oinas-Kukkonen and Harjumaa [10] introduced a social component, as well as individual use elements that organizations implement in practice. Similar to Oinas-Kukkonen and Harjumaa [10], we suggest that groups and social interactions play a key role in creating this ideal context for persuasive systems within work practices [12,13].

However, research continues and still focuses mainly on the individual [1]. Accordingly, we aim to address this gap in the literature by expanding the underexplored social aspect of persuasive technology. To do so, we rely on social cognitive theory [14,15] and a sociotechnical perspective to discuss the interconnections between social and technical aspects of improving users’ health with persuasive technology in organizational practice [11,16].

A recent publication with experiences from the field of practice [17] notes that people generally perceive benefits and costs when setting specific, measurable, attainable, realistic, and time-bound (SMART) goals in the context of home, work, and social influences. People equate individual level with better health outcomes, where some goals get aligned, and some goals do not. According to Burton-Jones and Grange [18], using a tool or system effectively requires cycling through the concepts of actions, consequences, disturbances, perceptions, goals, comparisons, and feedback, which is consistent with SMART goals; however, it provides more concepts in the iterations to help explain different patterns [19].

Previous research has primarily considered dyads of technology-mediated interactions within practice settings between patients and health care professionals (HCPs) [1]. There are other relevant interactions at play beyond the traditional patient–HCP dyad, notably when a platform is introduced within work environments. In such a context, the patient is interacting not only with HCPs but also with a range of other stakeholders, including insurers, supervisors, colleagues, family, friends, and mentors, where, arguably, platform use can influence all these interactions. We know that information technology can change how people interact at work [20], and when everyone in a work environment can access the same persuasive technology platform [9], it can generate conversations beyond the traditional dyads. The influence of these interactions can either reinforce or discourage system use in practice. We anticipate that these work-related factors will be especially salient when good health is an employment requirement.

### Setting

A prime example of a work environment that prioritizes good health is a fire station, which is a practical setting. We use this environment as our research setting for several reasons. First, firefighting requires good physical and mental health, and one might assume that firefighters are generally in excellent health to meet these demands of the work. Firefighters’ individual perceptions of benefits and effective use are well described in the literature [18]. However, cardiac deaths account for the largest proportion of deaths of firefighters each year [21], and rates of mental health problems are elevated for Canadian firefighters [22]. Moreover, although fitness and wellness standards have been recommended, only 30% of the US fire departments implement programs targeting these standards. Within a Canadian context, firefighters have higher than average body strength but are comparable with the general population in terms of aerobic capacity [23]. As such, firefighters would likely benefit significantly from using a health platform to improve their aerobic capacity. Perhaps most startling is the fact that firefighters have a 50% chance of developing cancer in their lifetime, possibly because of compounds within the smoke they breathe [19]. Thus, within these work environments, there is a strong need to focus on wellness that is integrated with medical care, family, work, and social systems, which we do in this paper.

## Methods

### Ethics Approval

The study was approved by the National Research Council – Industrial Research Assistance Program - Research Ethics Board.

### Recruitment

We collected data using an embedded qualitative case study design. The embedded case study facilitates the simultaneous consideration for multiple subunits of analysis, which for our purposes were the individual, group, and organizational. Specifically, we considered 8 fire station organizations, each composed of groups and individuals. Stations were randomly assigned to 2 treatment groups of either interactive webpages or static webpages [24,25]. Data consisted of document review and interview data presented with quotes [24,25]. We gained participation from 94.6% (141/149) of the firefighters.

Firefighters from a small Canadian city were selected because, in an initial meeting, they confirmed their need for increased physical activity and general health improvements, would participate in the study, and would help tailor content to their firefighter terminology; these met our selection criteria. Participation in the study required completion of wellness interventions at various points, willingness to use the platform, and openness to exploring collaborative relationships between leaders (eg, fire chiefs, human resource (HR) managers, union representatives, and mentors) and workers using the persuasive technology platform.

### Site Selection

The 1 embedded case study in a city with 8 fire stations allowed assignment of the stations to 2 treatment conditions, which were interactive persuasive technology webpages or static webpages, with half the stations (A, B, C, and D) randomly given interactive pages and the other half the stations (E, F, G, and H) given static pages. Fire stations received different combinations of nutrition, exercise, lifestyle, and medical goal-setting and advice on the persuasive technology platform over the 8-month study period. The research design data shown in Table 1 are best practices seen in the stations using interactive pages and absent in the stations using static pages.

**Table 1:** Research design for the study treatment program.

Description	Fire stations A, B, C, and D	Fire stations E, F, G, and H
Information sessions, account setup, and chili kickoff meeting (N=149)	77	72
Study treatment	Interactive webpages condition	Static webpages condition
Start baseline medical assessments (n=141)	73	68
End medical assessments (N=110)	57 using the webpages	53 using the webpages
If requested, received baseline prescription (N=53)	26 entered trackers at the beginning	27 entered trackers at the beginning
Interactive dashboard on webpages (N=73)	Interactive SMART^a^ goals, to-do action items, encouragements, blogs, RSS^b^ latest information feed, individual self-management, and extended access to HCPs^c^	None
Noninteractive dashboards on webpages (N=68)	None	Basic static content, basic self-tracking on paper, and basic information about interacting with persuasive technology platform
Nutrition and sodium awareness^d^	Interactive	Basic
Exercise step^d^ (N=19)	Interactive	Basic
Exercise bike^d^ (N=19)	Interactive	Basic
Dietitian^d^	Interactive—Canada Food Guide and DASH^e^ diet and salt-reduced diet	Basic—Canada Food Guide and DASH diet and salt-reduced diet
Behavior change	Interactive	Basic
Smoking cessation^d^ (N=21)	Interactive	Basic
Weight management	Interactive	Basic
Exit interviews (N=29)	Yes	Yes

^a^SMART: specific, measurable, attainable, realistic, and time-bound.

^b^RSS: Really Simple Syndication (web-based XML feed).

^c^HCP: health care professional.

^d^Group activity.

^e^DASH: Dietary Approaches to Stop Hypertension (for diabetics; reduced-sodium).

### Timeline and Procedures

**Figure 1:**
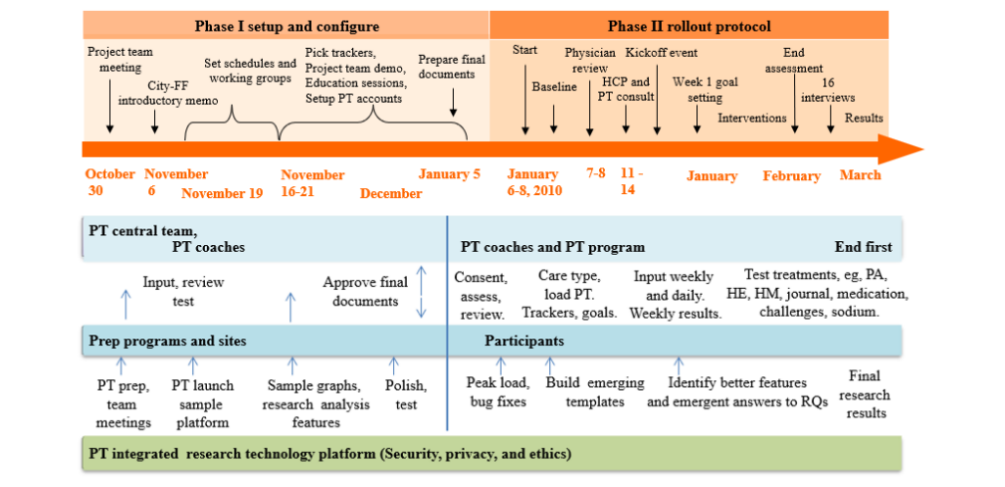
Timeline of research design events. FF: firefighter; HCP: health care professional; HE: healthy eating; HM: health management; PA: physical activity; PT: persuasive technology; RQ: research question.

The entire study period of 8 months was divided as follows. First, the phase I setup took 4 months, and phase II rollout proceeded over 4 months. It was followed by a maintenance (phase III) for 2 years after the study. As shown in Figure 1, the phase I setup had adaptable planning and approval for clinical practice guidelines that occurred in training and rehearsal of the framework proposed by Oinas-Kukkonen and Harjumaa [10]. The nature of firefighters’ tasks allowed us to apply the design principles identified by Oinas-Kukkonen and Harjumaa [10] to the professionals’ tasks in professional roles, which included HCPs, organizational HR professional, firefighters’ health expert, firefighters exercise expert, privacy officer, and stop smoking expert. The reduction of concepts (principle 1) fit because professional judgments were reduced to key points and directions based on pretesting, then the tunneling of points was strengthened to focus on ignoring other messages and tunneling to messages that professionals gave (principle 2), and the tailoring (principle 3) and personalization (principle 4) of messages for patients allowed professionals the freedom to be responsible for their decisions, to adapt the content and to create draft program page or pages, documents for weekly issue management, and frequently asked question help sheets in development [10]. The research team participated in pretesting of instruments in a formal rehearsal and reviewed the stations assigned to experimental study combinations.

The kickoff event for firefighters and HCPs had a 94.6% (141/149 firefighters participated) participation rate and engaged all participants from all 8 fire stations to follow at least one of the persuasive technology platform activities.

Medical assessments generally involved the physician overseeing the nurse capturing medical baselines for all participants, the other HCPs interacting with the firefighters, and the technology team (3/8, 38% to 3/15, 20%) answering questions and getting firefighters signed into their platform accounts.

To conduct the study, in addition to recruiting research participants, we recruited a 25- to 36-person *study team* that included 7 HCPs: 1 emergency room physician, 1 heart program nurse, 2 dietitians, 2 certified personal trainers, and 1 lifestyle mentor. Some of the other professional roles on the study team fell under an organization team (N=10-14), which included 1 union representative, 1 HR representative, and 8 to 12 organizational governance authorities, depending on the volume of activities with firefighters. The study team further included a technology team (N=8-15) of 5 to 12 technology development roles (depending on program pages being loaded), 1 data steward, and 2 HR consultants qualified to deliver the smoking cessation, stress, and general wellness guidelines.

**Figure 2:**
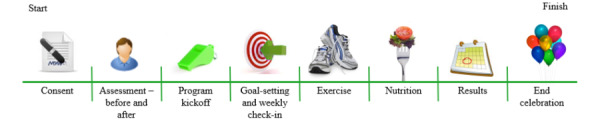
Process of goal-setting in phase II (rollout protocol timeline).

All 141 participating firefighters were offered possible individual goals on a private webpage that individuals would only see if they chose to use the individual page through the persuasive technology platform. Half the stations (4/8, 50%) had interactive pages (A, B, C and D), and half (4/8, 50%) had static basic pages (E, F, G, and H). In the interactive rollout, goals followed the SMART guidelines (ie, they were specific, measurable, attainable, realistic, and time-bound) [22] that were designed to increase behavior change for both individuals and groups. Nutrition information was based on either the reduced-sodium Dietary Approaches to Stop Hypertension *(DASH)* diet for hypertension [26-28] or the Canada Food Guide. The DASH diet is popular for sodium awareness given by the dietitian and heart disease advice given by the nurse. The static basic pages had general messages to stay healthy and eat well. Figure 2 illustrates the order of activities in the study.

All participants (N=141 at the beginning of the study and N=110 at the end of the study, using the webpages) were also offered possible group goals for group exercise step challenges, group exercise bike challenges, general nutrition (eg, reducing sugary drink intake), group sodium awareness, dietitian-recommended meal plans, weight management, and smoking cessation programs. The HCPs and organizational professionals helped firefighters establish overall group goal targets and group weekly wellness tasks via ongoing monitoring and blogging on webpages.

### Analysis Framework

We used qualitative case-based analysis [24,25], which included simple tallies in addition to thematic analysis. The data analysis generated evidence about complex interactions among individuals, groups, and work and social settings alongside HCPs, systems experts, and health measures using fun activities.

Thematic coding was implemented using NVivo software (QSR International) [29] to develop themes and identify patterns. The qualitative analysis involved multiple iterations of coding and comparing and compiling results. We created tallies and tables to demonstrate changes. Coding was done by 1 systems researcher and then checked by the technology and organization teams.

Ultimately, we compiled transcribed notes, compared patterns, and determined how many participants had succeeded (or failed) in reaching goals. We used the 11 factors identified by Orji and Moffatt [6] in their persuasive technology classification and analysis coding scheme to structure analysis, as shown in Table 2. Other persuasive technology studies presented these types of evidence.

**Table 2:** Persuasive technology classification scheme by Orji and Moffatt [6].

Factor	Identification	Description
1	Targeted (health) domain	Outcome changes for physical activity, healthy eating, medical issues, sleep, and hydration
2	Technology platform	Persuasive technology platform integrated web, mobile phone, weight scales, wearables, sensors, and watches
3	Duration of evaluation	Hours, days, weeks, months, and 2 years (a few people)
4	Behavior theories	Social cognitive theories with confidence and ability to do work tasks, goal-setting theory with interventions, self-management theory, performance evaluation theories, and processes improvement change theories
5	Motivation strategies	SMART^a^ goals, professional protocols, and intervention strategies; start anywhere, make changes for life, and any improvement matters
6	Evaluation method	Mixed methods comparing qualitative text-based, quantitative small sample size, log analysis, website feature use, and interviews
7	Targeted age group	≥18 years
8	Number of participants	8 fire stations with 141 people
9	Study country	East Coast, Canada
10	Targeted behavioral or psychological outcome	Integration of social cognitive abilities, behaviors, attitudes, adherence, mentoring behaviors, and social interactions
11	Findings or results	Whether successful overall and smaller changes during the paths that the users took

^a^SMART: specific, measurable, attainable, realistic, and time-bound.

**Figure 3:**
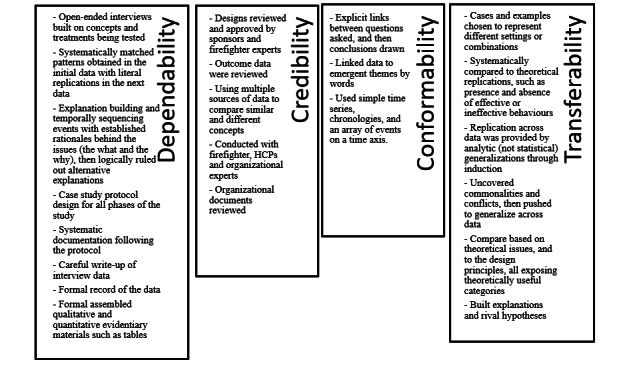
Mechanisms implemented to ensure research quality of data and analysis. HCP: health care professional.

Given that this research study relied on health assessments (taken by health professionals) and interview data from participants, we used appropriate methods to analyze the data, notably the qualitative data [30]. Specifically, we used analytic induction, reflexivity, triangulation, and member checks to convey aspects of trustworthiness of the research in terms of dependability, credibility, confirmability, and transferability as ways to assess the quality of qualitative research [31]. As shown in Figure 3, the dependability of the study is based on the logical, traceable, and documented nature of the embedded case design. The credibility of the research is based on using a multidisciplinary research team, drawing on multiple data sources and methods to triangulate the findings, while reaching theoretical saturation for our key themes. We also used member checks with participants to reflect our interpretations of the situation and whether they were reasonable assessments of what happened. The conformability is an indication of the link between the findings and interpretations to the data, which we have synthesized in Tables 3-6, whereby researcher reflexivity was used to identify themes and relationships among the themes and demonstrate better design principles and practices in persuasive systems and technology, adding to a growing body of related work. The transferability is illustrated by demonstrating similarities and differences between this study and previous work and theoretical and practical contributions made.

**Table 3:** Exemplars: operationalization of constructs for primary task support (design principles 1 to 7).

Design principle	Implementation of design principle in this study
Reduction: a system that reduces complex behavior into simple tasks helps users perform the target behavior, and it may increase the benefit-cost ratio of a behavior (principle 1).	Goal-setting for a wide range of key goals, including nutrition, exercise, and lifestyle changes (sleep, hydration, smoking cessation, weight loss, etc). Overall project outcomes set at the beginning to have fun, manage medical benefits costs better, improve health and wellness, manage cancer risks from exposure to fires, and gather evidence for work safety. Provide a private environment for personal discussions to occur. Access to resources and interactions that firefighters cannot easily coordinate themselves, such as interactive advice with HCPs^a^.
Tunneling: using the system to guide users through a process or experience provides opportunities to persuade along the way (principle 2).	Care plans developed as a result of a medical assessment by a physician and nurse and embedded in the persuasive technology platform to ensure the firefighters received appropriate care plans according to their medical conditions. The nurse identified firefighters with heart stents and updated the platform and coaches to alter the exercise suggestions.
Tailoring: information provided by the system will be more persuasive if it is tailored to the potential needs, interests, personality, use context, or other factors relevant to a user group (principle 3).	The programs were tailored by HCPs and other organizational professionals to fit firefighter needs.
Personalization: a system that offers personalized content or services has a greater capability for persuasion (principle 4).	A comprehensive set of measures were included for individuals to select exercise, nutrition, medical, and lifestyle trackers. The personalized measures could be chosen from exercise (light, moderate, and vigorous converted by the platform), walking, steps, running, strength training (sets, reps, and weight), fruits and vegetables, grain products, meat and alternatives, milk products, total sodium, total fiber, water, sleep, cigarettes per day, blood pressure, 4 cholesterol groups (total cholesterol, LDL^b^, HDL^c^, and ratio of TC^d^/HDL) and triglycerides, blood glucose, hip and waist measurements, height, weight, and a few other trackers that were not used in this paper (well-being, positive attitude, and medications taken). Any personalized measure could be refused or turned on at any time. The platform offered personalized advice based on their specific situation and progress. Individuals could release their trackers on their individual private webpage or release their trackers into a group challenge through a release feature. Users released with anonymous numbers or full names and could hide or rerelease data at any time in the future when they wanted. They had individual control.
Self-monitoring: a system that helps track one’s own performance or status supports in achieving goals (principle 5).	Self-management controlled by individual firefighters with their own unique username and password; they had individual pages with personal summary information of their trackers (measures), goals set with medical or persuasive technology staff, group challenges to join, or lifestyle changes to make. Individuals could enter data at any time and choose to share their data or keep it private.
Simulation: systems that provide simulations can persuade by enabling them to observe immediately the link between the cause and its effect (principle 6).	None.
Rehearsal: a system providing means to rehearse a behavior can enable people to change their attitudes or behavior in the real world (principle 7).	Rehearsal with experts within the firefighters’ organizations (eg, union representatives, station mentors, and chiefs) ensured approval and quality before the start, as well as consistency and thoroughness. The researcher prepared and pretested protocols with the professional team to prove the accuracy of the platform operation. Rehearsal for firefighters occurred with account log-in demonstrations and privacy options during the first interaction—the medical assessment day.

^a^HCP: health care professional.

^b^LDL: low-density lipoprotein.

^c^HDL: high-density lipoprotein.

^d^TC: total cholesterol.

**Table 4:** Exemplars: operationalization of constructs for dialogue support (design principles 8 to 14).

Design principle	Implementation of design principle in this study
Praise: by offering praise, a system can make users more open to persuasion (principle 8).	There were weekly check-ins via the persuasive technology platform with HCPs^a^ and other organizational professionals who offered praise for accomplishments and encouragement to increase goals. The platform facilitated “walking buddies” (pairs) who could encourage regular walks and wellness discussions.
Rewards: systems that reward targets may have great persuasive powers (principle 9).	The platform gave digital rewards, such as trophy icons, on the fly through HCPs (eg, chats and email). Winning teams were listed on leaderboards. Learning from 1 healthy breakfast offered through the platform to persuade other stations. Dietitians offered improvements around healthy pancake breakfast for a first station and went to stations to illustrate the changes in their cupboards, fridges, and group breakfasts.
Reminders: if a system reminds users of their target behavior, the users will more likely achieve their goals (principle 10).	To their individual private webpage, users received weekly results and reminders of their individual trackers (measures), goals, and interactions (summarized from users’ primary tasks, which they were given by HCPs). The platform presented group averages, which individual firefighters could review whenever they wanted.
Suggestion: systems offering suggestions at opportune moments will have greater persuasive powers (principle 11).	The platform produced a professional dashboard with graphs where HCPs reviewed graphs to identify which users needed new suggestions (eg, a personal trainer could post new tailored exercises). Other suggestions were planned for firefighters who plateaued and stopped progressing as suggested in care plans. In weekly reviews, the HCPs devised new suggestions at the exact time they saw an issue on dashboard graphs. Hence, interventions happened quickly. HCPs also watched for gaps in the evidence and the absence of questions from firefighters to offer more suggestions. A lack of questions could indicate plateauing, and advisers watched to probe if firefighters wanted more suggestions.
Similarity: people are more readily persuaded through systems that remind (them of) themselves in some meaningful way (principle 12).	Content tailored to the firefighters’ wellness documents, their union’s wellness terminology, and experts’ suggestions. HCPs and other organizational experts connected terminology to other national standards (eg, hypertension and privacy standards) to the content on webpages.
Liking: a system that is visually attractive for its users is likely to be more persuasive (principle 13).	The look and feel of the platform were designed to be visually appealing and easy to navigate and used gamification principles where possible.
Social role: if a system adopts a social role, users will more likely use it for persuasive purposes (principle 14).	Stations mentors, coaches, personal trainers, persuasive technology account helpers, and HCPs took on social roles (principle 14) to advise other firefighters and begin dialogues. Once dialogues started, then the social dialogue management moved into suggesting principle 11.

^a^HCP: health care professional.

**Table 5:** Exemplars: operationalization of constructs for system credibility support (design principles 15 to 21).

Design principle	Implementation of design principle in this study
Trustworthiness: a system that is viewed as trustworthy (truthful, fair, and unbiased) will have increased powers of persuasion (principle 15).	Curated content was written by professionals to fit national protocols and approved by firefighter-sanctioned expert groups (ie, nutrition, exercise, and union wellness standards).
Expertise: a system that is viewed as incorporating expertise (knowledge, experience, and competence) will have increased powers of persuasion (principle 16).	Professional certifications were important to ensure firefighters followed the advice and engaged in the activities. The HCPs^a^ also worked primary jobs that lent credibility to their expertise, which included the physician who worked in the local hospital emergency room, the nurse who worked in heart program clinics, the personal trainers who owned popular local gyms, and the dietitians who worked in local health system practices. The platform was built by the technology company, primarily led by the research director who designed pleasing interfaces, and the persuasive technology was then managed by the developers for back-end technical infrastructure, database, and security. Privacy and confidentiality were managed by the research director. The professionals’ content was given to the tech company, which staged the content, rehearsed with professionals, and integrated with study wellness goals. The physician and nurse medical protocols were demonstrated in development-type use cases to be compliant with Health Level 7 tracker and terminology standards.
Surface credibility: people make initial assessments of the system credibility based on a firsthand inspection (principle 17).	The platform pages were designed to allow trials and demonstrations. The interface, flow, and navigation shown in the initial information session received positive feedback.
Real-world feel: a system that highlights people or organizations behind its content or services will have more credibility (principle 18).	The platform connected trackers for work and home lifestyle changes to manage the more difficult behavior changes (eg, lack of sleep, overeating, substance abuse, and mental health issues). The platform adapted to requests for individuals or groups to work on something, which included trying to foster family lifestyle changes.
Authority: a system that leverages roles of authority will have enhanced powers of persuasion (principle 19).	All content and pages were approved by station chiefs, union, city government, and sponsors for funding of the study. Sponsors only received aggregated results and had no access to individual trackers (measures), groups, or comments. Reports were summarized into anonymous personas or group averages.
Third-party endorsements: third-party endorsements, especially from well-known and respected sources, boost perceptions of system credibility (principle 20).	Platform trackers (measures) and group challenges fit the union representative’s expectations, and specific sections in the international standard wellness document were noted on the bottom of webpages.
Verifiability: credibility perceptions will be enhanced if a system makes it easy to verify the accuracy of site content via outside sources (principle 21).	Presented comparable information to the Canada Food Guide for general nutrition advice and DASH^b^ salt-reduced diet suggestions for reduced-sodium diets when required (eg, for patients with heart disease or high-risk participants).

^a^HCP: health care professional.

^b^DASH: Dietary Approaches to Stop Hypertension (for diabetics; reduced-sodium).

**Table 6:** Exemplars: operationalization of constructs for social support (design principles 22 to 28).

Design principle	Implementation of design principle in this study
Social learning: a person will be more motivated to perform a target behavior if he or she can use a system to observe others performing the behavior (principle 22).	The persuasive technology platform presented chats on the landing page (eg, chat with a dietitian or whichever professional was logged in at the time). It also presented social walls such as Facebook functionality, which were optional and internal to the software that were used to create a social environment and friend one another, discuss similar interests, and get to know users and their family information.
Social comparison system: users will have a greater motivation to perform the target behavior if they can compare their performance with the performance of others (principle 23).	The platform presented group averages for step challenge and bike challenge, where comparisons could be made privately on individual viewing pages or shared in group challenge pages where users’ data would be visible under the name they chose (real full name, real short name, pseudonym fake name, or anonymous).
Normative influence: a system can leverage normative influence or peer pressure to increase the likelihood that a person will adopt a target behavior (principle 24).	The platform presented national medical norms within a table on users’ individual page, where their personal individual medical assessment and trackers were compared with national norms (by age, gender, and race) for weight, blood pressure, cholesterol, and so on. Medical assessments were done at baseline and the middle and end of the study. Individuals could track their progress against averages for the entire sample.
Social facilitation: system users are more likely to perform target behavior if they discern via the system that others are performing the behavior along with them (principle 25).	The platform presented a “trash talk” discussion board for the bike exercise challenge where firefighters exchanged fun discussion and egged each other on. Group challenges were done with group workouts in stations.
Cooperation: a system can motivate users to adopt a target attitude or behavior by leveraging human beings’ natural drive to cooperate (principle 26).	All programs were designed to build social comparisons into them with friendly encouragement in cooperative ways and not too competitive. The smoking cessation program paired smokers with a nonsmoking buddy to alter at-work smoking behavior and offer social interaction when they wanted to smoke. This social support worked because smokers indicated they wanted social interaction at work; the evidence shows that smoking is not good for them, but many smoke, anyway.
Competition: a system can motivate users to adopt a target attitude or behavior by leveraging human beings’ natural drive to compete (principle 27).	Six challenges were offered (ie, step, bike, sodium awareness, nutrition generally, smoking cessation, and weight management).
Recognition: by offering public recognition (for an individual or a group), a system can increase the likelihood that a person or group will adopt a target attitude or behavior (principle 28).	Group challenge winners were celebrated with digital rewards and persuasive technology platform webpage announcements. Kickoff celebration offered healthy chili recipe.

## Results

In this section, we review the results that interact across multiple levels, starting with a brief overview of individual results and then focusing on study results that extend the current understanding by examining groups of people using a platform (ie, examining social and work contexts).

### Individual Integrated Self-management Behaviors

Our results for individuals achieved many positive health and wellness impacts similar to those of other studies [1], where individuals improved medical, exercise, nutrition, and lifestyle trackers. In the final interviews (N=29), 51% (15/29) of participants stated that they viewed the persuasive technology platform as a positive catalyst for their wellness behavior change and 37% (11/29) individuals lost an average of 7.3 kg. Although many stated they already had good wellness behaviors, it was unclear what that meant in practice. Notably, the platform had a stronger influence on those who did not have an established routine (ie, an average of 60% score on behavior change measure; N=29) compared with those who did (ie, an average of 44% score on behavior change measure; N=29).

### User Journeys

#### Overview

For each user story, results are generally organized by level of analysis, starting with the individual, then the group, and finally the organization level. Within each level, we first consider key themes and link those to important outcomes that each user story illustrates.

Patterns began to emerge that illustrated differences in individuals’ holistic outcomes—that is, outcomes captured at every level of an individual’s life (N=141 at the beginning of the study and N=110 at the end of the study, using the webpages). For example, it cannot be considered a positive move if someone stops smoking at work only to take up drinking to manage the stress of quitting cigarettes. Thus, we sought to capture combinations that accounted for such possibilities. In order to convey those fast and flexible combinations, we mapped the 28 design principles identified by Oinas-Kukkonen and Harjumaa [10] onto our constructs, as shown in Tables 3-6.

Among these various user journeys, several achieved better patterns using interactive webpages but also showed that integrated change compared with the design principles needs to be made carefully with much adaptability to local situations (N=141 at the beginning of the study and N=110 at the end of the study, using the webpages). Qualitative case evidence shown in Tables 3-6 provide data to support these claims that demonstrate the better practices seen under interactive webpages (ie, stations A, B, C, and D), which were absent when compared with patterns under static webpages (ie, stations E, F, G, and H). The evidence data are exemplars of better design principles; they are not exhaustive. Overall, our results showed that systems and platforms yield far better results when integrated with support from society, families, colleagues, social settings, and organizations, which begins some evidence of this integration.

In the user journeys, evaluation of platform use patterns (along the principles described in Tables 3-6) are presented along with outcome evaluations by professionals. All evaluations were performed on a none, poor, fair, good, great, or excellent scale. These show linkages between use of features following the design principles of persuasive technology and outcomes.

#### Individual User Journey 1: Tom (Poor Platform Use Pattern, Leading to Fair Health Outcomes; Influencers: Vague Wellness Objectives and Few Good Success Concepts; Orthotics Barriers)

One person that the persuasive technology platform helped was Tom, a firefighter in his mid-40s, paid by the city, who had been dealing with some physical ailments that created challenges with exercise. Tom was a prime example of a user who set vague wellness objectives, not as specific as other firefighters who set SMART goals and who achieved higher outcomes. Tom had high computer skills but did not push himself very hard to improve. Sleep apnea was preventing him from getting the proper rest he needed to fuel his 2-job lifestyle (he had a second job in construction on weekends). Time was also a barrier for exercise; however, he remained active at least three days a week. Tom used his pedometer to diligently track and record his own steps, deliberately challenging himself. He watched the group challenge, without actively participating, by logging in to the system as a spectator but was hesitant to join the actual activities. Tom needed new orthotics, and to him, that was a substantial barrier to lower body training and his exercise goal of gaining muscle; this made it impossible for him to track strength training efforts. Because exercise caused joint discomfort, he only walked each day, missing much of the moderate exercise asked of him. He reviewed program software pages and read educational material but did not engage much further when others began to adopt new behaviors. Tom zeroed in on general nutrition information in the software and got a push in the right direction, managing to lose 10 lbs. The biggest barriers for Tom were lack of time and complex physical and medical barriers. Orthotics were ordered in the hope that this would address his lack of exercise at the moderate levels asked by the HCPs.

We coded the user journey of Tom as *poor* according to the primary task support principles (principles 1 to 7) identified by Oinas-Kukkonen and Harjumaa [10] because he did not reduce the concepts well, had little pathway tunneling, had little tailoring (general vague wellness objectives, not specific as SMART goals suggest), had little personalization (focused on broader, general program pages), had some self-monitoring for his own steps but little else (eg, step challenge, sodium reduction, and strength exercises), had no simulation or trying new things, and had little rehearsal with others in the group [10]. Hence, overall, Tom underperformed, even though any positive change could be viewed as a success and was celebrated with him, when comparing across patterns, he missed these many behaviors that others adopted to reach higher or better outcomes as seen in the next journeys.

#### Group Follower User Journey 2: Harvey (Lurker Not a Participant, Fair Platform Use Pattern, Leading to Fair Health Outcomes: Typical Individual Wellness Patterns but Lacking Significant Improvement; Influencers: Avoiding Group Platform Activities)

The persuasive technology platform also helped Harvey, a user who typified those who followed groups but never participated in group activities. The introduction of groups allowed individuals to join organizational groups or to ask work friends to join them. In this study, they chose to place themselves together in groups (eg, firefighters, union representatives, chiefs, and HR department) [32]. These emergent groups could then challenge other groups and join other groups.

Similar to many other participants, Harvey had a great general nutrition and exercise regime, wanted to get blood work done, and addressed a previous high cholesterol issue. His nutrition had improved before the program when a family member discovered they were hypertensive and was maintained in the average range throughout the program. He was concerned about family wellness and lack of time.

Harvey followed the bike challenge by monitoring the group results on the program page but did not participate. However, he reported not being able to find his medical results, which were on the same program page. Harvey was also looking for additional ways to destress. He had a good grasp on his balanced lifestyle but wanted new ways to incrementally improve his approach. He achieved this by making a small shift in his nutrition, exercise, and sodium awareness, hence leading to minor improvements. He confirmed that his cholesterol levels had shifted within the normal range. We coded the user journey of Harvey overall as *fair* according to the primary task support principles (principles 1 to 7) around broader, general wellness goals identified by Oinas-Kukkonen and Harjumaa [10] because he was only watching the social activities, not participating in or performing the other behaviors, which was principle 22 (social learning), and was mostly not well engaged in activities such as the others were.

#### Group Challenge User Journey 3: Allen (Excellent Platform Use Pattern, Leading to Excellent Health Outcomes; Influencers: Joined Too Many Groups, but Fun Groups Were His Organizing Mechanism)

Fun group challenges available through the persuasive technology platform included excellent participants such as Allen in the sodium reduction, steps, bike exercise, and general nutrition programs (Table 6; all principles 22-28). Allen did the many group activities described here, as the exemplar excellent group participant. He liked to access HCPs’ chats and blogs, which were well received (Table 5; principles expertise and surface credibility). The group challenges were appreciated because they delivered integrated engaging social, group pages, while allowing individualized medical advice to be seen only on individual private screens (Table 3, primary task [all]; Table 4, rewards; Table 5, real-world feel, not a demo; and Table 6, normative influence and recognition). The personalized individual timing (Table 3; personalization) kept pace with public and fun group activities (Table 3; tailoring) [9].

The overall results reveal an immediate change in shopping behaviors and a 73% change in sodium awareness (N=29), as well as a 59% change in overall nutrition awareness which Allen achieved. Actual nutrition intake improved by 22% on average (N=29 interviewed of 94.6% (141/149) using webpages at the beginning and 73.8% (110/149) using webpages at the end. We purposely combined individual, group, and social concepts that fit the setting to capture the complex work and home interactions that existed.

The program further motivated firefighters such as Allen with a fun kickoff event game called *guess the salt*, which included access to content and features on the persuasive technology platform through direct knowledge from experts and appealing interactive pages. This approach was used to manage the user patterns systematically without too much drill-down that becomes fatiguing for HCPs. The firefighters indicated in the final interviews (N=29) that the game’s well-designed graphics helped them change their behaviors and achieve their excellent results. After the kickoff event, the program in parallel conducted tailored and personalized baseline assessments for individuals (Table 3; tailoring and personalization) [33,34] in a private setting close to work activities (N=141 at the beginning of the study and N=110 at the end of the study, using the webpages). This approach helped employees, employers, and professionals improve more easily [7,9].

Group programs [35] were offered around achievable social challenges [3,14,16] chosen by firefighters such as Allen, who showed excellent platform use patterns (eg, exercise, nutrition, sodium awareness, and smoking cessation and led by certified professionals). These groups combined two or more people in to (1) engage the power of group check-in and persuasion, (2) identify a wellness challenge goal or objective that all group members wanted to meet [32-34,36], and (3) pick specific measures [33,36] that participants could record on paper and in the persuasive technology platform, which was then converted into the shared effort on the platform.

The program also had weekly behaviors [35] that firefighters, including Allen, recorded for weekly group results charting, chatting between people or leader competitions. It set end points 6-8 weeks from the start to give closure and motivation. End celebrations [36] included digital rewards or physical prizes from the organization. The maintenance phase III continued for 2 years to identify sustainable behaviors for life [10,17] beyond the typical 2-month falling-off point when participants stop good behaviors and go back to their starting point. In parallel and in an integrated manner, the program managed professional oversight dashboards [6] to deliver program information [5] and identify when an intervention was needed to the few exceptions by the professionals’ known patterns of participation.

Group nutrition behaviors were shaped by program design options such as the fun sodium and nutrition challenges, which involved all of the participants attending a dietitian-led interactive sodium awareness lecture that Allen enjoyed (N=149), followed by discussion, label reading introduction, and 2 months with features on the persuasive technology platform (N=141 at the beginning of the study and N=110 at the end of the study, using the webpages). The dietitians advised compliance with the Canada Food Guide (69/141, 48.9%) and Dietary Approach to Stop Hypertension low-sodium diet (72/141, 51.1%; numbers provided by a dietitian) with identified actionable behaviors such as *your next trip to the grocery store* and immediate ways to remove unwanted sodium.

In general, group exercise awareness increased by 55% (average 5.5/10 ratings for 19/29, 63%), and the most frequent comment from participants such as Allen was that they were “more consistent in filling the periods of inactivity with exercise” (quote from the interview). Among those who participated in the group step and bike challenges, 65% (19/29) of the individuals reported results from their participation [23].

We observed evidence of exercise avoidance among the moderate to low exercisers who did investigate exercise and nutrition information via the platform but did not push themselves hard or consistently. We defined low to moderate exercisers as those who exercised, on average, 3 days a week, for 1.10 hours at a time, at a 4.46 intensity level (lower), and within more general workouts. These are much lower than the intense exercisers, who we defined as those who exercised, on average, 4.23 days per week, for 1.09 hours at a time, and at a 7.32 intensity level (high). Our findings suggest that persuasive technology platforms should offer a wide variety of choices to sway moderate to low exercisers.

#### Distrusted Organization User Journey 4: David (Good Platform Use Pattern—Once Trust Issues Were Addressed and There Were Good Health Outcomes, He Stopped Smoking; Influencers: Required Privacy Shield to Continue in the Smoking Cessation Program Group Because He Distrusted the Organization Work Environment for Demonstrated Reasons; Well Organized by Nonsmoking Buddy at His Fire Station Work Environment)

##### Overview

The *stop smoking* program enlisted 21 firefighters, including David, at the initial assessment (evidence counted), which resulted in good outcomes for David but tempered by distrust of the work organization. These participants chose to conduct the programs on the platform as a group social challenge. However, when it came time to participate in the stop smoking program, only 62% (13/21) of the firefighters confirmed their participation. David was one of the participants who might have backed out, so we use him as an example of distrust toward the organization and to explore what it took to get him through.

##### Distrust in the Work Environment

Many firefighters, including David, hesitated to use employer wellness resources and medical benefits that would reveal that they smoked, as they believed that would allow their employer to classify them as individuals who engaged in unhealthy behaviors and even fire them. The firefighters reported stories about other firefighters losing a promotion or being let go for smoking.

As related evidence, no firefighter took up the offer from the HR manager to cover CAD $150 (US $118.5) of nicotine patches as a medical benefits payment. The distrust of HR by David resulted in concealing unhealthy behaviors from his employer, as he believed, such as many of the firefighters, that this exposure would threaten his career. Thus, one perceived downside of incorporating health platforms at work is a worry that health conditions could be misinterpreted by employers as weaknesses on the job, hindering or halting career development.

##### Privacy Shield Design Element

The technology company quickly developed a *privacy shield* to address the distrust by David and to allow all firefighters confidential access to the smoking cessation resources. The firefighters also confirmed the need for anonymity as important.

##### Credible Book or Information Source

As a consequence of all these forces, the smoking cessation program needed credible content behind the privacy shield to be successful (Table 5; principle of surface credibility), which David appreciated. The first component was the *Easy Way to Stop Smoking* book, chosen by a third-party consultant to ensure it was appropriate for an organizational environment. The persuasive technology platform anonymously averaged the number of cigarettes not smoked for the group.

##### Nonsmoking Social Buddy System

The other component that contributed to the success of the smoking cessation program was introducing each user to a nonsmoking coworker (ie, social nonsmoking buddy), which we propose further reinforced the social group effect and a displacement effect with healthier wellness behaviors. Several firefighters reported they stopped smoking on their own or with the help of the family physician after discussions that took place in the program. Notably, however, they went directly to a physician, not using the patch offered by HR—an indication of their ongoing distrust of the organizational work environment.

#### Valued Discussions in the Workplace User Journey 5: Jack (Fair Platform Use Pattern, Leading to Great Health Outcomes; Influencers: Access to HCP Advice but Not Group Activities)

##### Overview

Rich and complex work-related discussions occurred that highlighted the challenges associated with the pursuit of health initiatives in work environments (N=141 at the beginning of the study and N=110 at the end of the study, using the webpages; N=29 interviewed at the end of the study). In some cases, when health issues are directly related to the work environment, even the narrow use of the platform can lead to significant improvements. One particular work-integrated medical assessment outcome discussion between the nurse and one of the firefighters, Jack, is used as an illustration. We use the term *great work-related discussion* to encompass those rich settings beyond the initial, typical discussions between the HCPs and participants (detailed further in the next section). In this way, the persuasive technology platform and program had the potential to reveal information or behaviors unknown to the organization, which entailed work implications beyond other influences such as the details provided in the next section.

##### Effective Work-Integrated Assessment Outcomes

Firefighters considered the blood work screenings to be work-related activities because of their aforementioned increased occupational risk of cancer. This danger motivated many firefighters, including Jack, to undertake the platform challenges and individual activities to get markers of clean blood or evidence of contaminated blood. To them, tasks such as blood work represented long-term, effective, work-related evidence for occupational hazards and a way to manage their work-related health concerns.

For Jack, the middle and end medical assessments showed improved medical outcomes, where he specifically valued medical outcomes evaluated in the professional work context that improved for him and many others. Firefighters such Jack felt that it was improved workplace access to HCPs (such as the nurse), who were more aware of specific work-related options available to the firefighters, that motivated them to achieve these great results, which were comparable with recommended national norms (N=141 at the beginning of the study and N=110 at the end of the study, using the webpages).

#### Work-Related Professional User Journey 6: Nurse (Excellent at the Start but Failing Toward the End, Platform Use Pattern; Influencers: Excellent HCP Work but Fell Back to Silos)

Although firefighters perceived medical assessments as meaningful for individual medical outcomes or group averages, HCPs perceive medical assessments as a normal, typical part of professional health care work [5]; this created different perceptions that influenced the integrated persuasive technology platform use. Hence, we examine how HCP work roles fall back into silos if they are not reworked into holistic team-based care along with the principles (Table 5).

##### Excellent Work-Related Medical Assessment Process and Primary Tasks in the Work Setting of Practice (Nurse)

The nurse completed 53 blood work requisitions for the 141 people at the baseline assessment, which appear as typical HCP work with physical measurements of participants, and then assessed medical and family histories. Considered by itself, the medical baseline, middle, and end look similar to excellent but regular nurses’ work.

##### Slippage From Integrated Work Roles Back to Siloed Work Roles

The persuasive technology platform systematically delivered blood work results to each firefighter and provided an internal email from the physician and nurse for medical assessment onto a private individual webpage.

The study integrated HCPs’ work well for the work setting where the physician’s and nurse’s approved protocols and prescriptions were coordinated and individualized blood work options, relevant to the medical history of Jack and clinical conditions that met both professions’ protocols [5].

#### Organization and Program Manager Results With Reasonable Costs

The user stories illustrate several interesting patterns, which suggest that everyone values the services that was done at a reasonable cost. Statements about values included “oversight of entire program progress gave greater comfort, ease of program setup eased the burden of preparing, consent and messages gave less to manage and destroy, engaging fun group activities, improving wellness overall” (ie, increase exercise, nutrition, and sodium awareness), and “implementing joint union-mandated wellness programs.” These quotes match social and technical concepts that build on and improve the quality of the persuasive technology platform.

The reasonable costs were achieved at a fraction of the cost of other technologies and activities. The persuasive technology wellness challenge was licensed at approximately CAD $12,000 (US $9480) in the first year to operate the challenge and avoided CAD $17,613 (US $13,914.27) in other general wellness and disease costs for this small municipal local government organization. The total cost savings include CAD $6913 (US $5461.27) of general wellness cost saving calculated by improvements for wellness program outcomes for the number of firefighters multiplied by savings per year identified in the literature. Outcomes for the increase in physical activity, smoking cessation, weight loss and maintenance, and improvements in nutrition were multiplied by appropriate cost numbers noted in literature and then added to form a total. The rest of the cost savings was CAD $10,700 (US $8453) for disease cost savings calculated in a similar way from acute conditions better managed (eg, high blood glucose, back pain, muscle pain, sleep apnea, hypothyroidism, stroke, high cholesterol, high blood pressure, gastrointestinal ailments, asthma, colitis, kidney issues, chronic obstructive pulmonary disease, and depression) [8,28].

## Discussion

### Principal Findings

The results of this study confirm recent findings [1] that persuasive technology can alter individuals’ outcomes and behaviors through frequent interaction beyond the initial contact points with the technology. By studying the use of a persuasive technology platform in a work environment, we found that the combined, integrated influences of work, group, social, and individual outcomes reinforce one another through this platform. In this way, we illustrate an interconnected social and technical system—but it is one that needs far more work to implement holistically in team-based care settings for life.

### Individual Outcomes

As expected, the persuasive technology platform [1] improved firefighters’ individual outcomes on several fronts. There was dramatic improvement in sodium awareness (29/141, 20.6%) with a reported 73% improvement on our awareness scale, 37% (11/29) of individuals lost an average of 7.3 kg, and a medical baseline (N=141) was established with medical issues identified for blood pressure, total cholesterol, low-density lipoprotein cholesterol, blood glucose, and other conditions.

The firefighters in our study were motivated by these processes and perceived benefits. They interacted with programs as individuals and as colleagues in groups, whereas professionals created the material to make the program happen. Consistent with previous findings [24], we observed that using feedback to compare consequences with goals generally led to more effective use of the platform and perceived benefits. Individuals engaged more with the persuasive technology platform’s features when HCPs’ work tasks were integrated into the use of those features through the platform and the firefighters’ work and social networks (N=141 at the beginning of the study and N=110 at the end of the study, using the webpages). This finding was confirmed during final interviews. All design and execution elements of the study had to focus on benefits for successful behavior change to achieve the great number of improvements seen in the study.

### Organizational Work Outcomes

The persuasive technology platform created an environment that motivated firefighters to have more health- and wellness-related conversations at their organizational work, even though it did not always lead to exercise-tracking behavior. We suggest that a repeatable persuasive technology platform with sustainable programs, deployed in an organizational work setting, produced these combined health improvements. The platform changed work behaviors, which station mentors observed as higher numbers of participants discussing exercise around the stations and asking more specific wellness questions. Work discussions with friends, coworkers, and HCPs increased greatly but had to fit within the organizational workplace certification requirements and be adapted to meet union guidelines. In some settings, peer and supervisor influences can prevent technology use if they do not like that technology. Given that firefighters eat together while on the job, there was much discussion with dietitians about how to change station group meals. Firefighters developed a stronger understanding of their own health through talking directly to dietitians at work.

The studied organizational work environment—such as any work environment—was not neutral, and this hindered participation. There was a level of distrust between the employer and employees in this organization and certain information that employees did not want shared with their employer. The smoking cessation program revealed the need for a secure *privacy shield* for these in-depth organizational health and wellness programs to succeed. The fact that some behaviors or conditions could eventually be made visible to the employer was a significant concern for participants.

At the same time, as there are many common elements in the individual participants’ conditions, the organizational work environment could be very well suited to offer support and well-targeted interventions by HCPs. In our study, professional assessments were adjusted to consider the unique features of the professional work environment. The study helped align multiple health care protocols and checked for possible work-related diseases. For some participants, significant medical issues were uncovered that would not have been detected as early without the program and without the persuasive technology platform in the organizational work setting (29/53, 55% of the prescriptions were out of range, where 15/29, 52% were serious medical issues). Firefighters were not visiting their physicians regularly, and this was made visible by the persuasive technology platform.

It is puzzling that even in an organizational work environment where poor health could lead to loss of employment, we still found that participants struggled to develop healthy behaviors and observed unproductive workplace wellness messages. In this sense, firefighters represent a critical case, and the observations from this environment can serve as important examples for other organizations.

### Groups Offer Fun, Social Distractions at Work Around Confidential, Serious Work-Related Health Issues

Group components, which represented the HCPs’ tasks in the programs they ran, were designed to support participants’ work processes and became meaningful tasks for the individual firefighters. Goals were set for individuals and for groups, which increased motivation and changed behaviors. Individuals were motivated by the desire to not disappoint the group. This motivation was especially salient with the exercise, sodium reduction, and smoking components. Ultimately, the study’s comprehensive program achieved many outcomes around exercise improvements, smoking cessation, management of serious medical issues, and improvements in general nutrition (N=29 final interviews; N=141 at the beginning of the study and N=110 at the end of the study, using webpages).

In this way, group influence went beyond the motivational component to embed in individuals’ tasks and organizational work processes. Discussions with work colleagues around health are likely to contribute to a better understanding of the data presented in a persuasive system and of the expected consequences of actions. This increased understanding is likely to lead to appropriate action [18]. Evidence from the discussions among the participants supports this pattern. Hence, we argue that integrating social components of groups in organizations with persuasive technology and systems increases the potential benefits of these platforms.

### Better Integrating the Social, Work, and Technical Environments

The interplay among these persuasive technology platforms, work, and personal interactions enabled the success of the programs and fostered the firefighters’ willingness to continue after the study ended: at the end of the 8-month study period, the firefighters asked the systems researcher to establish additional studies through the HR department and emergency room physician, which she designed and handed to the organization. These participants acknowledged that they could not have figured out the design or executed it at the high level that was achieved in the study.

Platform use helped users gain control over their health conditions throughout iterations, leading to improved health and wellness after several weeks. The persuasive technology platform made efforts visible to the group and reminded individuals that their respective efforts were contributing to a larger group or social goal. Seeing the progression of results helped participants persevere toward their goals with the added motivation, as noted, of not disappointing their peers. This result is consistent with research in other contexts where interfaces provided feedback about performance and led to increased group performance through social reinforcement [35].

The repeatable persuasive technology platform gave participants integrated, personalized medical advice and personalized intervention oversight on a large, systematic scale. The study’s professionals had to work within organizational settings, which included HCPs in team-based care that was facilitated by the technology, through fast and flexible approaches. The end result provided firefighters with a comprehensive approach that accounted for the complexity of the local situation, integrating individual, group, work, social, home, and professional influences.

### Path Forward

This paper argues that health and wellness improvements occurred in this study because the organization and participants involved used a persuasive technology platform that was designed to reflect its use in work, group, home, and social systems beyond initial interactions. The persuasive technology platform enabled employees to reveal behaviors such as smoking, drinking, or reckless driving behind a privacy shield, which probably would not have occurred without this protection, and then change those behaviors. In alignment with the 28 principles identified by Oinas-Kukkonen and Harjumaa [10] for designing persuasive systems, we allowed people to disclose sensitive data to subsequently improve their situation. Tables 3-6 provide a useful illustration of how the features of an implementation reflect 96% (27/28) of the design principles [10].

We present a vision for personal health record platforms that considers the wider improvements that could be made in understanding health decisions, addressing participants’ motivation, and facilitating meaningful use of the technology in the individual users’ local work setting. These persuasive technology platforms fundamentally offer a different approach to encourage new social interaction through work and home environments beyond the initial interactions. Given that a significant portion of the provincial government budgets in Canada already pay for the health system and many people still lack primary health care [37-39], these holistic platforms and team-based HCP care at a reasonable cost should be embedded in many organizational work settings.

### Limitations and Future Research

The fact that our data were drawn from 1 Canadian city limits our ability to directly compare our context with others with different characteristics. For instance, large urban areas may make it harder to create strong groups if the social fabric is not as tightly woven as it is in smaller communities. Because of data aggregation, we are unable to provide findings by smaller groupings, which limits our results. Furthermore, we intentionally conducted this study in a context where good health is an essential job requirement.

However, it would be interesting to see whether these results are replicated in the same manner in settings where health is not seen as essential for the work performed; this could be a boundary condition for the findings observed. Our findings illustrate that workplace health and wellness are inextricably linked with what happens with the individual and the group both on and off the job. Any sustainable persuasive technology that links workers with professional care teams must navigate the work versus personal life combined with individual versus group dynamics.

## Abbreviations

DASH: Dietary Approaches to Stop Hypertension
HCP: health care professional
HR: human resource
SMART: specific, measurable, attainable, realistic, and time-bound
